# Modulation of cortisol responses to the DEX/CRH test by polymorphisms of the interleukin-1beta gene in healthy adults

**DOI:** 10.1186/1744-9081-7-23

**Published:** 2011-07-05

**Authors:** Daimei Sasayama, Hiroaki Hori, Yoshimi Iijima, Toshiya Teraishi, Kotaro Hattori, Miho Ota, Takashi Fujii, Teruhiko Higuchi, Naoji Amano, Hiroshi Kunugi

**Affiliations:** 1 Department of Mental Disorder Research, National Institute of Neuroscience, National Center of Neurology and Psychiatry, Kodaira, Tokyo, 187-8502, Japan; 2 Department of Psychiatry, Shinshu University School of Medicine, Matsumoto, 390-8621, Japan; 3 Department of Medical Genetics Majors of Medical Sciences, Graduate School of Comprehensive Human Sciences, University of Tsukuba, Tsukuba, 305-8577, Japan; 4 Core Research of Evolutional Science & Technology (CREST), Japan Science and Technology Agency (JST), Tokyo, 102-0075, Japan

## Abstract

**Background:**

Recently, hypothalamus-pituitary-adrenal (HPA) axis function assessed with the combined dexamethasone (DEX)/corticotropin releasing hormone (CRH) test has been shown to be associated with response to antidepressant treatment. A polymorphism (rs16944) in the interleukin-1beta (*IL-1β*) gene has also been reported to be associated with the medication response in depression. These findings prompted us to examine the possible association between *IL-1β *gene polymorphisms and HPA axis function assessed with the DEX/CRH test.

**Methods:**

DEX/CRH test was performed in 179 healthy volunteers (45 males: mean age 40.5 ± 15.8 years; 134 females: mean age 47.1 ± 13.2 years). Five tagging single nucleotide polymorphisms (SNPs) of *IL-1β *gene (rs2853550, rs1143634, rs1143633, rs1143630, rs16944) were selected at an r^2 ^threshold of 0.80 with a minor allele frequency > 0.1. Genotyping was performed by the TaqMan allelic discrimination assay. A two-way factorial analysis of variance (ANOVA) was performed with the DEX/CRH test results as the dependent variable and genotype and gender as independent variables. To account for multiple testing, *P *values < 0.01 were considered statistically significant for associations between the genotypes and the cortisol levels.

**Results:**

The cortisol levels after DEX administration (DST-Cortisol) showed significant associations with the genotypes of rs16944 (*P *= 0.00049) and rs1143633 (*P *= 0.0060), with no significant gender effect or genotype × gender interaction. On the other hand, cortisol levels after CRH administration (DEX/CRH-Cortisol) were affected by gender but were not significantly influenced by the genotype of the examined SNPs, with no significant genotype × gender interaction.

**Conclusions:**

Our results suggest that genetic variations in the *IL-1β *gene contribute to the HPA axis alteration assessed by DST-Cortisol in healthy subjects. On the other hand, no significant associations of the *IL-1β *gene polymorphisms with the DEX/CRH-Cortisol were observed. Confirmation of our findings in futures studies may add new insight into the communication between the immune system and the HPA axis.

## Background

Recent studies have provided several lines of evidence implicating the pro-inflammatory cytokine interleukin-1beta (IL-1β) in the etiology and pathophysiology of depression. Studies investigating peripheral levels of IL-1β have reported elevated concentrations of IL-1β in patients with major depression [1-3] and dysthymia [4,5]. In experimental animals, exogenous administrations of IL-1β have produced depressive-like symptoms, which were attenuated by treatment with antidepressants [6,7]. Furthermore, administration of the IL-1 receptor antagonist has ameliorated the stress-like effects in cellular and behavioral models [8,9], making it a possible candidate for therapeutic targets.

A few studies have examined genetic polymorphisms of the *IL-1β *gene for association with depression. Although no significant allelic or genotypic differences have been found between patients with depression and healthy controls [10-12], three studies have consistently shown that the G allele of rs16944 in the *IL-1β *gene is associated with poor response to antidepressant treatment [10,13,14]. This evidence suggests that genetic regulation of inflammatory processes mediated by IL-1β is involved in the pathophysiology of depression and resistance to antidepressant treatment.

Alterations in the hypothalamic-pituitary-adrenal (HPA) axis have been well-documented in depression and related psychopathological conditions (reviewed by [15,16]). We have also reported heightened HPA axis in major depression and its normalization after treatment as assessed with the combined dexamethasone (DEX)/corticotropin releasing hormone (CRH) test [17,18]. The relationship between alteration of the HPA axis activation and immune alterations in depression has been described in several studies [19-21]. The increased expression of pro-inflammatory cytokines is thought to lead to the progression of the immune response and activation of the HPA axis [22-24]. In particular, IL-1β is considered to play an important role in the HPA axis alteration. A significant positive correlation between mitogen-induced IL-1β production and post-DEX cortisol values have been reported, suggesting that IL-1β hypersecretion may contribute to HPA axis hyperactivity [25]. Animal studies have further demonstrated that the release of CRH stimulated by IL-1 is involved in the HPA activation [26-28].

Intriguingly, a number of studies have shown that the DEX/CRH test may be a predictor for response to antidepressant treatment. Ising et al [29] found that higher cortisol level after DEX administration prior to CRH stimulation was associated with a favorable treatment outcome. Two recent studies showed that response to antidepressant treatment was more favorable in depressive patients whose peak cortisol levels during the DEX/CRH test were reduced after the initiation of the treatment [30,31]. Further, in the study by Binder et al [32], antidepressant response was associated with higher cortisol response to the DEX/CRH test in male, but not female, patients. It is plausible that the DEX/CRH test and the antidepressant response are related, since antidepressants may exert their clinical effects, at least in part, by reducing the activity of the HPA system [33,34]. The fact that both the HPA axis activity and the *IL-1β *gene polymorphism influence the response to antidepressants further supports the evidence of reciprocal relationships between the HPA system and IL-1β.

These previous findings prompted us to examine the possible association of polymorphisms in the *IL-1β *and the HPA function. In the present study, the relationship between the *IL-1β *gene polymorphisms and the results of the DEX/CRH test was examined in healthy subjects. Because gender effects on measures of HPA axis function have been previously reported [32,35], a two-way factorial analysis of variance (ANOVA) was performed with the DEX/CRH test results as the dependent variable and genotype and gender as independent variables to determine the possible interaction effects between the polymorphisms and gender.

## Methods

### Subjects

Subjects were 179 adult healthy volunteers (45 males: mean age 40.5 ± 15.8 years, 134 females: mean age 47.1 ± 13.2 years) recruited from the community through advertisements in free local information magazines and by our website announcement. Most of the subjects were from our previous sample, in which the relationship between stress, sleep, and HPA function was examined [36-38]. All subjects were biologically unrelated Japanese individuals without current or past history of psychiatric treatment, and were screened with a direct contact interview by a research psychiatrist using the Japanese version of the Mini International Neuropsychiatric Interview (M.I.N.I.) [39,40] to rule out any axis I psychiatric disorders. Participants were excluded if they had prior medical histories of central nervous system disease or severe head injury, if they met the criteria for substance abuse or dependence or mental retardation, if they had received glucocorticoid treatment, psychotropic treatment, antihypertensive medications, oral contraceptives, or estrogen replacement therapies in the previous month, or if they suffered from any inflammatory, infectious, or systemic immune diseases, based on self-reports, at the time of assessment. The study protocol was approved by the ethics committee at the National Center of Neurology and Psychiatry, Japan. After description of the study, written informed consent was obtained from every subject.

### Genotyping

**Figure 1:**
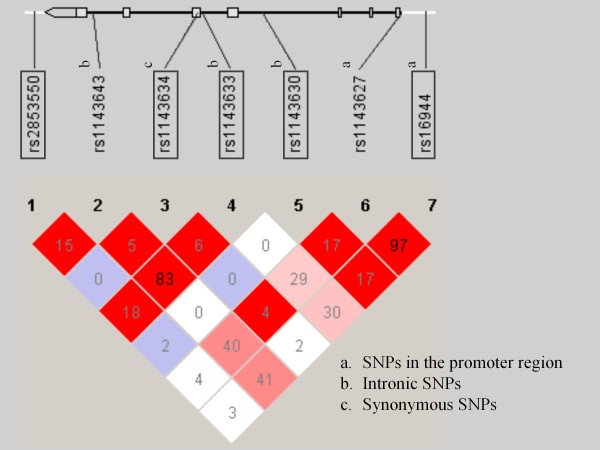
**Genomic organization and LD structure of the *IL-1β *gene**. Genomic organization and linkage disequilibrium (LD) structure of the *IL-1β *gene are shown. Exons are shown as boxes. Shades of pink represent extent of LD (red denotes D' = 1). Numbers in squares give r^2 ^values multiplied by 100. The names of the examined SNPs are enclosed in boxes.

Five tagging single nucleotide polymorphisms (SNPs) in a region 1 kilobase (kb) upstream to 1 kb downstream of the *IL-1β *gene (rs2853550, rs1143634, rs1143633, rs1143630, rs16944) were selected by Haploview 4.2 [41] using Japanese and Chinese population in the HapMap SNP set (release 22), at an r^2 ^threshold of 0.80 with a minor allele frequency greater than 0.1. Genomic organization and linkage disequilibrium structure of the *IL-1β *gene are shown in Figure 1. Genomic DNA was prepared from the venous blood according to standard procedures. The SNPs were genotyped using the TaqMan 5'-exonuclease allelic discrimination assay. Thermal cycling conditions for polymerase chain reaction were 1 cycle at 95°C for 10 minutes followed by 50 cycles of 92°C for 15 seconds and 60°C for 1 minute. The allele-specific fluorescence was measured with ABI PRISM 7900 Sequence Detection Systems (Applied Biosystems, Foster city, CA, USA). Genotype data were read blind to the DEX/CRH test results. Ambiguous genotype data were not included in the analysis.

### DEX/CRH test

The DEX/CRH test was administered according to a protocol proposed in a previous report [18], which was also employed in our recent report [36]. The subjects were administered 1.5 mg of DEX (Banyu Pharmaceutical Corporation, Tokyo, Japan) orally at 23.00 h. On the next day, they attended our laboratory and sat on a comfortable couch in a calm room. A vein was cannulated at 14.30 h to collect blood samples at 15.00 and 16.00 h via an intravenous catheter. Human CRH (100 μg) (hCRH 'Mitsubishi', Mitsubishi Pharma Corporation, Tokyo, Japan) was administered intravenously at 15.00 h, immediately after the first blood collection. Subjects fasted and rested semi-supine throughout the testing. Blood samples were immediately centrifuged and stored at -20°C. Plasma concentrations of cortisol were measured by radioimmunoassay at SRL Corporation (Tokyo, Japan). The detection limit for cortisol was 1.0 μg/dl. Cortisol values under the detection limit were treated as 0 μg/dl. The intra-assay coefficients of variation at 2.37 μg/dl, 13.02 μg/dl, and 36.73 μg/dl were 6.90%, 4.94%, and 5.78%, respectively. The inter-assay coefficients of variation at 2.55 μg/dl, 13.04 μg/dl, and 34.17 μg/dl were 8.91%, 6.03%, and 6.44%, respectively (SRL Corporation, Tokyo, Japan). Outcome measures of this neuroendocrine test were the DST-Cortisol (i.e., the concentration of cortisol [μg/dl] at 15.00 h) and DEX/CRH-Cortisol (i.e., the concentration of cortisol at 16.00 h).

### Statistical analysis

Deviations of genotype distributions from Hardy-Weinberg equilibrium (HWE) were assessed using the χ^2 ^test for goodness of fit. To determine the interaction effects between genotype and gender, two-way factorial analysis of variance (ANOVA) was performed with the transformed cortisol levels as the dependent variable and genotype and gender as independent variables. Because the cortisol levels were not normally distributed, the aligned rank transformation method was used to transform the data prior to conducting ANOVA [42]. To correct for the multiple comparisons, statistical significance was set at two-tailed *P *< 0.01 when the analysis was performed for each of the 5 SNPs. Otherwise, statistical significance was set at two-tailed *P *< 0.05. Analyses were performed using the Statistical Package for the Social Sciences (SPSS) version 11.0 (SPSS Japan, Tokyo).

## Results

None of the examined SNPs deviated significantly from HWE after Bonferroni correction (all *P *> 0.01), although rs1143633 showed a trend towards excess of heterozygotes over Hardy-Weinberg expectations (χ^2 ^= 6.48, *P *< 0.05). Age showed no significant correlation with DST-Cortisol or DEX/CRH-Cortisol in either gender (all *P *> 0.05).

**Figure 2:**
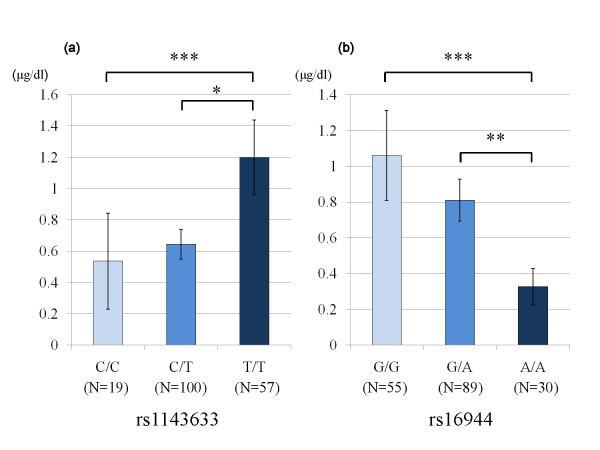
**Mean DST-Cortisol levels**. The means of the DST-Cortisol levels in each genotype of rs1143633 (Figure 2a) and rs16944 (Figure 2b) are shown. Error bars indicate the standard errors of the means. Asterisks in the graph indicate significance levels of the Tukey's post hoc test. * P < 0.01, ** P < 0.001, *** P < 0.0001.

Additional file 1 (Table S1) presents the results of the two-way ANOVA performed with the transformed plasma cortisol levels as the dependent variable and genotype and gender as independent variables. Significant effects of genotype on DST-Cortisol were observed for rs1143633 and rs16944, with no significant gender effect or genotype × gender interaction. The means of the DST-Cortisol in each genotype of rs1143633 and rs16944 and the significance levels of Tukey's post hoc tests are shown in Figure 2. Regarding DEX/CRH-Cortisol, significant gender effects were demonstrated in three of the two-way ANOVA results. No significant genotype effect or genotype × gender interaction was observed for DEX/CRH-Cortisol.

## Discussion

To our knowledge, this is the first study that revealed the possible role of *IL-1β *polymorphisms on HPA axis function. Two studies have reported the influence of genetic polymorphisms on DEX suppression test (DST) [43,44], one of which has shown that an allele in a polymorphism within the *CRHBP *gene, which regulates the CRH system, was associated with pronounced DEX suppression of corticotropin and with nonresponse to citalopram treatment in depressed subjects [43]. Some studies have also reported the influence of genetic polymorphisms on DEX/CRH-Cortisol. Schule et al [45] reported that *brain-derived neurotrophic factor *Val66Met polymorphism is associated with HPA axis activity during the DEX/CRH test in depressed patients. There is also a study reporting an association of a polymorphism of the *preprogalanin *gene with DEX/CRH test results and antidepressant treatment response [46]. Our study adds to accumulating evidence that genetic factors could influence the activity of the HPA axis.

Previous studies have shown associations of HPA axis activity assessed by the DEX/CRH test with antidepressant treatment [30-32]. Furthermore, higher cortisol level after DEX administration prior to CRH stimulation was also associated with a favorable treatment outcome [29]. Since the *IL-1β *gene polymorphism is also known to influence the response to antidepressants, we expected that association between *IL-1β *gene and HPA activity assessed by the DEX/CRH test would be found. However, our results showed that only DST-Cortisol, but not DEX/CRH-Cortisol, was significantly associated with the *IL-1β *gene polymorphisms. Thus, the relationship of the HPA activity and the *IL-1β *gene polymorphism with the antidepressant response could not be fully explained from our findings. Although the present study was performed in healthy subjects, it is possible that a different regulatory mechanism of HPA axis which overweighs the effect of the polymorphisms examined in the present study may exist in depressive patients. Further studies must be carried out to determine HPA axis regulation in patients with depression.

Significant gender effects were observed for DEX/CRH-Cortisol, consistent with previous studies [18,47]. However, as mentioned above, the DEX/CRH-Cortisol did not show significant associations with any of the SNPs examined. This may partly be explained by the putative action of IL-1β on hypothalamus. Animal studies have shown that IL-1β is involved in the noradrenaline-induced release of CRH from the hypothalamus [28,48]. Therefore, higher IL-1β expression in the brain may facilitate the release of CRH even when the suppressive effect of DEX is exerted, resulting in higher DST-Cortisol. On the other hand, the effect of IL-1β on the DEX/CRH-Cortisol could be small if the administration of CRH overweighs the effect of IL-1β expression level. Because rs1143634 and rs1143633 showed a trend towards association with DEX/CRH-Cortisol (*P *< 0.1), a further study with a larger sample size may be warranted.

The findings on biological roles of rs16944 polymorphism have not been consistent across studies. A/A genotype has been associated with higher gastric mucosa IL-1β levels in H. pylori positive population [49]. On the other hand, mononuclear cells from subjects with G/G genotype showed an increased release of IL-1β after stimulation with lipopolysaccharide [50]. Recent studies suggest that the functional role of rs16944 may depend on the *IL-1β *promoter region haplotypes including rs16944 and rs1143627 [51-54]. Although the findings are inconsistent, these previous studies suggest that rs16944 could affect the expression levels of IL-1β. The biological role of rs1143633, on the other hand, has not been previously reported.

Previous studies showed that administration of DEX inhibited lipopolysaccharide (LPS)-stiumulated production of IL-1β [55,56], suggesting that DEX may affect the function of *IL-1β *gene. Therefore, future studies should examine the influence of *IL-1β *gene polymorphisms on not only basal IL-1β levels but also DEX-induced IL-1β levels. The decrease of the LPS-induced *IL-1β *gene expression by DEX may be caused by the inhibition of nuclear factor binding to the *IL-1β *gene promoter [56]. It is noteworthy that, in the present study, the HPA response to DEX was affected by rs16944, which is located in the promoter region of the *IL-1β *gene.

The following limitations must be considered when interpreting the results. First, since the DEX/CRH test used here was based on a simple test protocol, we could not calculate the cortisol area under the curve [57,58]. We also did not measure the adrenocorticotropic hormone levels, which was measured in previous studies to determine the pituitary glucocorticoid negative feedback [30,45,57-59]. Moreover, we did not measure the baseline levels of cortisol. The extent to which the cortisol was suppressed in response to DEX administration could not be determined from our data. There may have been a genotype dependent alteration of the baseline cortisol levels, which appeared in the present study as a genotype effect on DST-Cortisol. This will need further investigation. We also did not perform physical examination to rule out physical disorders. As the presence or absence of inflammatory and immune diseases were based only on self-reports, the results may have been affected by unrecognized inflammatory processes in some participants. Furthermore, the lack of data on menstrual cycle or menopausal status in the female participants may have also influenced the results. Another limitation of the study is that we did not assess the biological role of *IL-1β *polymorphisms. The association of IL-1β expression level with the *IL-1β *polymorphisms needs to be investigated in the future. Finally, the present study included only healthy subjects. Whether the relationship between *IL-1β *gene polymorphisms and the HPA function is altered in depressed patients remain to be elucidated.

## Conclusions

The present study revealed that the cortisol response to administration of DEX is influenced by the *IL-1β *gene polymorphisms. The G allele of rs16944 and the T allele of rs1143633 were significantly associated with higher DST-Cortisol. On the other hand, no significant associations of the *IL-1β *gene polymorphisms with the DEX/CRH-Cortisol were observed. Our study adds to accumulating evidence that IL-1β is involved in the regulation of the HPA axis. Confirmation of our findings in futures studies may add new insight into the communication between the immune system and the HPA axis.

## Competing interests

The authors declare that they have no competing interests.

## Authors' contributions

DS and HK designed the study and DS wrote the draft of the manuscript. DS, HH, TT, KH, MO, and HK screened the study participants using the Mini International Neuropsychiatric Interview (M.I.N.I.). HH performed the DEX/CRH test. DS and YI performed the genotyping. DS, HH, and TF undertook the statistical analysis. HK supervised the data analysis and writing of the paper. TH and NA also supervised the writing of the paper and gave critical comments on the manuscript. All authors contributed to and have approved the final manuscript.

## Supplementary Material

Additional file 1 **Table S1. The results of the two-way ANOVA**. ANOVA was performed with the transformed cortisol levels as the dependent variable and genotype and gender as independent variables. P values < 0.01 are shown in bold. Click here for file
